# FeS_2_ Nanoparticles Encapsulated in S/N Co-Doped Carbon Nanofibers With a Three-Dimensional Multi-Channel Structure for Lithium-Ion Batteries

**DOI:** 10.3389/fchem.2022.957462

**Published:** 2022-07-15

**Authors:** Xiaochang Cao, Yi Zhang, Chujiang Luo, Yansheng Yin, Yingying Huang

**Affiliations:** ^1^ College of Mechanical Engineering, Dongguan University of Technology, Dongguan, China; ^2^ Institute of Science and Technology Innovation, Dongguan University of Technology, Dongguan, China; ^3^ Research Center for Corrosion and Erosion Process Control of Equipment and Material in Marine Harsh Environment, Guangzhou Maritime University, Guangzhou, China

**Keywords:** FeS_2_, carbon nanofibers, three-dimensional multi-channel structure, cathode material, lithium-ion batteries

## Abstract

Pyrite (FeS_2_) is one of the potential candidates for advanced rechargeable Li-ion batteries (LIBs) owing to its inherent capacity (849 mAh g^−1^), environmental friendliness, and abundant natural resources. However, the volume expansion of FeS_2_ and the dissolution of polysulfide in the electrochemical reaction severely limit its application in the field of energy conversion and storage. Herein, FeS_2_ nanoparticles are encapsulated in S/N co-doped three-dimensional multi-channel structural carbon nanofibers (FeS_2_@CNFs) through the electrospinning method. As a cathode material for LIBs, FeS_2_@CNFs demonstrated excellent rate property and cyclic stability. The 3FeS_2_@CNFs (weight ratio of FeS_2_ is 30%) present the initial capacity of 1,336.7 mAh g^−1^ and the remaining 856.5 mAh g^−1^ at 0.02A g^−1^ after 100 circles. The favorable electrochemical properties have confirmed that carbon nanofibers can enhance the electroconductivity of electrodes, reduce the volume collapse of FeS_2_, and remit the dissolution of polysulfide during the Li^+^ ions insertion/de-insertion process. In addition, co-doped S/N can supply abundant active sites for electrochemical reactions, providing enough space for Li^+^ ion storage. The results indicate that 3FeS_2_@CNFs is a cathode with a developmental prospect for LIBs.

## Graphical Abstract

Graphical Abstract Novel FeS_2_@CNFs nanocomposites with the multi-channel structure were successfully prepared using the electrospinning method. The three-dimensional interlinked multi-channel carbon nanofibers can facilitate the diffusion of Li^+^ ions and electrons. Meanwhile, the FeS_2_ nanoparticles are distributed on the inner wall of the carbon nanofibers, improving the phenomenon of volume expansion for FeS_2_ and preventing the dissolution of polysulfide during the cycling process. In addition, co-doped S/N can supply abundant active sites for electrochemical reactions, providing enough space for Li^+^ ion storage. The FeS_2_@CNFs and the preparation method have exceptional applications in the field of energy storage.
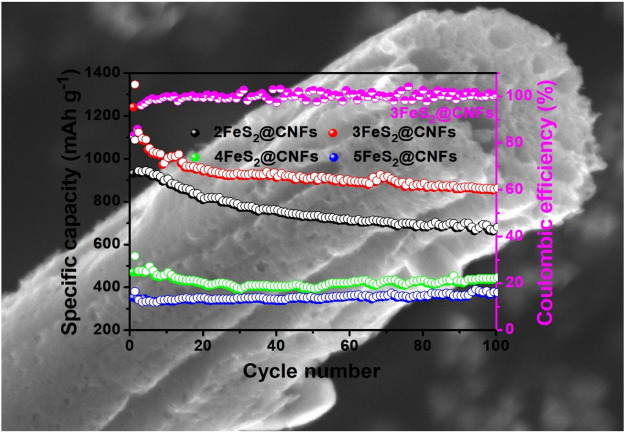

## 1 Introduction

As the population continues to increase, the energy demand is also growing rapidly (Zhao et al., 2015; Chi et al., 2018; Teng et al., 2019; Kesavan et al., 2020). The overexploitation of non-renewable fossil fuels has seriously polluted the environment (Zhang et al., 2019; Fang et al., 2021; Yang et al., 2021). Compared to traditional fossil fuels, electricity is a green, low-carbon, environment-friendly, and efficient energy system. To date, researchers have conducted numerous studies on electric energy storage. The common commercial electronic storage devices currently used contain nickel–cadmium batteries, lead–acid batteries, nickel–metal hydride batteries, Li-ion batteries (LIBs), fuel cells, etc. Among them, LIBs have been universally used in various fields such as manned crafts and small equipment because of their advantages of high energy density, excellent cyclic stability, and low self-discharge. Therefore, LIBs have attracted great attention. For instance, Gou et al. (2021) prepared Li_3_VO_4_/C through a facile agitation–drying method combined with subsequent calcination. The as-prepared composites were used as anode materials for LIBs and exhibited outstanding electrochemical properties. Zhong et al. (2018) synthesized a sandwich-type sulfur@Co/N-doped carbon ternary composite for Li–S batteries. The assembled Li–S batteries display excellent energy storage performance and provide the possibility of realizing industrially practical energy. Jiang et al. (2020) encapsulated NaTi_2_(PO_4_)_3_ nanoparticles in N/S dual-doped carbon (NTP@CNS) as the anode for LIBs via the sol–gel method followed by calcination treatment. The NTP@CNS shows excellent electrochemical property. According to daily needs and the rate of production, various types of LIBs are prepared. Recently, Li–FeS_2_ batteries are considered to be one of the power batteries having the most potential. However, the volume expansion of FeS_2_ during the intercalation and de-intercalation of Li^+^ ions lead to a structural collapse, reducing the cycle life of LIBs (Zhang et al., 2016; Wang et al., 2021). Meanwhile, the conversion process is accompanied by accessory substances such as lithium polysulfides (Li_2_S_*x*_, 2 < *x* < 8). These accessory substances can make the conductivity between the anode and current collector worse. In addition, the lithium polysulfides also dissolve in the electrolyte and can gradually migrate to the cathode, leading to an increase in the impedance of the cathode (Wang et al., 2019; Li et al., 2021).

To overcome these existing issues, researchers have also attempted to nanosize FeS_2_. Nanocrystallization can effectively alleviate the damage caused by Li^+^ ions insertion/de-insertion of active materials during the charge and discharge processes, improving the cyclic stability (Lei et al., 2016). Meanwhile, the nanoscale of active substances also effectively shortens the ion transmission path, accelerates the Li^+^ ions diffusion rate, and improves the rate property (Polishchuk et al., 2019). Li et al. (2014) reported the synthesis of phase-pure FeS_2_ nanowires through thermal vulcanization of the precursor *α*-FeF_3_ 3H_2_O nanowires. The nano-FeS_2_ cathode retained 350 mAh g^−1^ after 50 circles at 0.1°C. Liao et al. (2013) fabricated macroporous FeS_2_ nanotubes through a solvothermal method. The macroporous FeS_2_ nanotubes exhibited 925.2 mAh g^−1^ and retained 439 mAh g^−1^ at 0.2°C after 60 cycles. Nevertheless, the preparation of nanostructured single-phase FeS_2_ has long-term challenges due to the presence of many substoichiometric Fe–S phases and orthorhombic FeS_2_ (Ennaoui et al., 1993). Therefore, researchers began to attempt to hybrid nanostructured FeS_2_ with carbon materials. Carbon materials can not only improve the electrical conductivity and relieve the volume expansion of electrodes but also delay the damage of polysulfides during charge and discharge processes (Xu et al., 2016; He et al., 2017). For instance, Xu et al. (2016) synthesized a FeS_2_@HPC composite through the formation of FeS_2_ nanocrystals in hierarchical porous carbon. The as-fabricated FeS_2_@HPC presented 907 mAh g^−1^ and maintained 720 mAh g^−1^ after 100 circles at 1°C. Xu Q.-T. et al. (2018) prepared the biomass-carbon@FeS_2_ composites from auricularia auricula after the carbonization and sulfidation procedure. The as-synthesized composite demonstrated 850 mAh g^−1^ after 80 circles at 0.5°C. Wang et al. (2021) reported a raspberry-like hierarchical-structured FeS_2_ cathode modified by the dual-carbon framework. The as-prepared cathode delivered 566 mAh g^−1^ and maintained a capacity reduction rate of 0.014% for each circle at 1°C. These studies demonstrate that the development of nanocomposites combining FeS_2_ with carbon can improve the electrochemical properties of electrodes.

Herein, a type of FeS_2_@carbon nanofiber (FeS_2_@CNF) nanocomposites with a multi-channel structure was successfully prepared using the electrospinning method. The three-dimensional interlinked multi-channel and S/N co-doped carbon nanofibers can improve the electroconductivity of cathodes. Meanwhile, the lotus-like structure can ameliorate the phenomenon of volume expansion for FeS_2_ and prevent the dissolution of polysulfide during the cycling process. The effect of the FeS_2_ content on properties was studied through examining the performances of FeS_2_@CNFs nanocomposites with different contents of FeS_2_. The application feasibility of FeS_2_@CNFs as cathodes for LIBs was also explored in detail.

## 2 Experiment

### 2.1 Material Preparation

**FIGURE 1:**
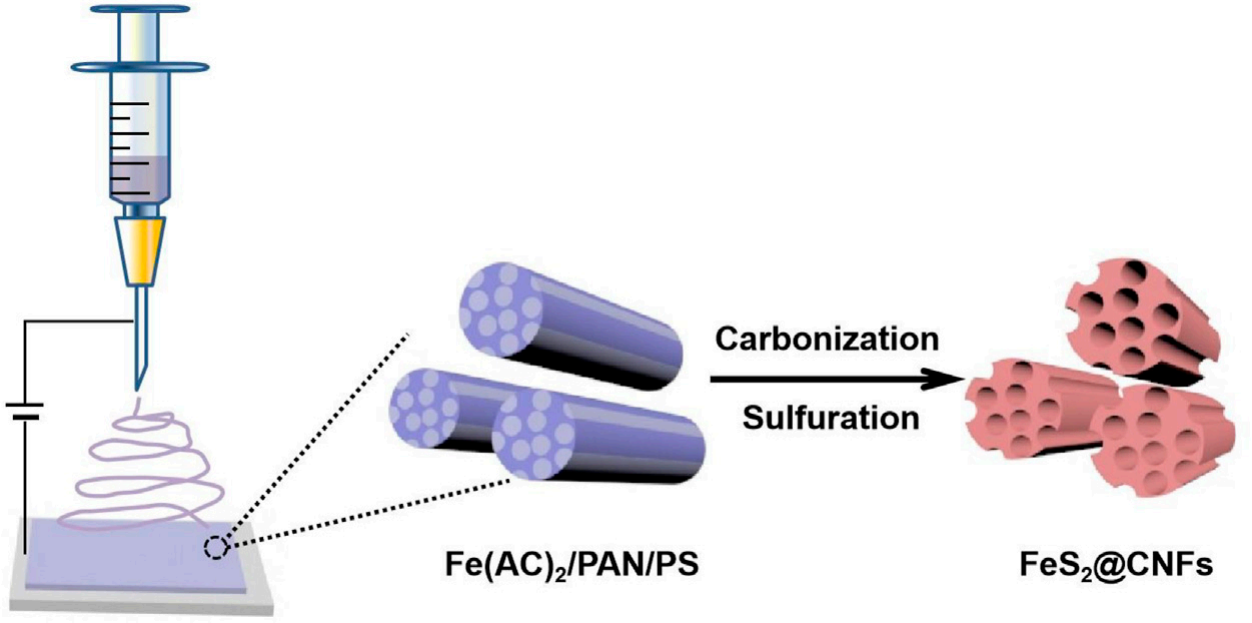
Illustration of the procedure for the preparation of FeS2@CNFs.

A total of 340 mg iron acetate, 400 mg polystyrene, and 500 mg polyacrylonitrile (PAN) were poured into 5 mlN, N-dimethylformamide and mixed at 65°C for 12 h. The aforementioned mixture was then electrospun with a single nozzle (21 gauge needle). The distance between the syringe and the receiver was 15 cm, the voltage was 17 kV, and the injection rate was 1 mlh^−1^. The as-prepared precursor film was stabilized at 200°C for 2 h and then calcined at 800°C with 5°C min^−1^ for 4 h in an Ar/H_2_ atmosphere. After reducing to 30°C, the film was sealed with sulfur powder in a quartz tube (V_product_: V_sulfur_ = 1:2). Subsequently, the quartz tube was heated to 600°C and kept for 6 h. After that, the product was dissolved in CS_2_ to eliminate redundant sulfur. Finally, it was dried in vacuum at 100°C to obtain a lotus root–like FeS_2_@CNFs with many channels. The preparation process of FeS_2_@CNF nanocomposites based on the electrospinning approach is illustrated in Figure 1. The content of FeS_2_ in FeS_2_@CNFs nanocomposites prepared by this process was 20 wt%, which was named 2FeS_2_@CNFs. Samples with contents of 30, 40, and 50 wt% were also synthesized in the same way and named 2FeS_2_@CNFs, 3FeS_2_@CNFs, 4FeS_2_@CNFs, and 5FeS_2_@CNFs, respectively.

### 2.2 Characterization

The crystal structure information was obtained on a Rigaku diffractometer with Cu K*α* radiation (*λ* = 1.5418) within 10–90°. Raman measurements were performed on an HR800 spectrophotometer from 400 cm^−1^ to 2400 cm^−1^. The information of the valence states was acquired using a Thermo ESCALAB 250 X-ray photoelectron spectrometer (XPS) with monochromatic Al K*α* (1486.6 eV). The surface morphologies were observed using scanning and transmission electron microscopes (SEM, Ultra Plus, ZEISS and TEM, Talos F200X). The SEM was obtained at 10 kV. TEM was acquired at 200 kV accelerating voltage.

### 2.3 Electrochemical Measurements

The synthesized FeS_2_@CNFs nanocomposites were directly used as the cathodes of LIBs without any conductive agent, binder, and metal collector. The film of FeS_2_@CNFs was cut into a circle with a diameter of 1 cm. The mass of each cathode was about 1 mg cm^−2^. A total of 1 M LiPF_6_ in a mixture of vinyl carbonate/dimethyl carbonate (1:1 in volume) was used directly as the electrolyte. Lithium disks were used as the anode, and the Celgard 2400 microporous polypropylene membrane was employed as the separator. The aforementioned materials were assembled into CR2032 coin-type cells in an argon-filled glovebox and tested for electrochemical properties. The electrochemical properties were tested by using a CHI760E workstation and a Land CT 2001A battery testing system. The cyclic voltammetry (CV) and the galvanostatic charge and discharge (GCD) performances were determined between 1.0 and 3.0 V. Electrochemical impedance spectroscopy (EIS) was conducted at the frequency of 10^5^–10^–2^ Hz.

## 3 Results and Discussion

**FIGURE 2:**
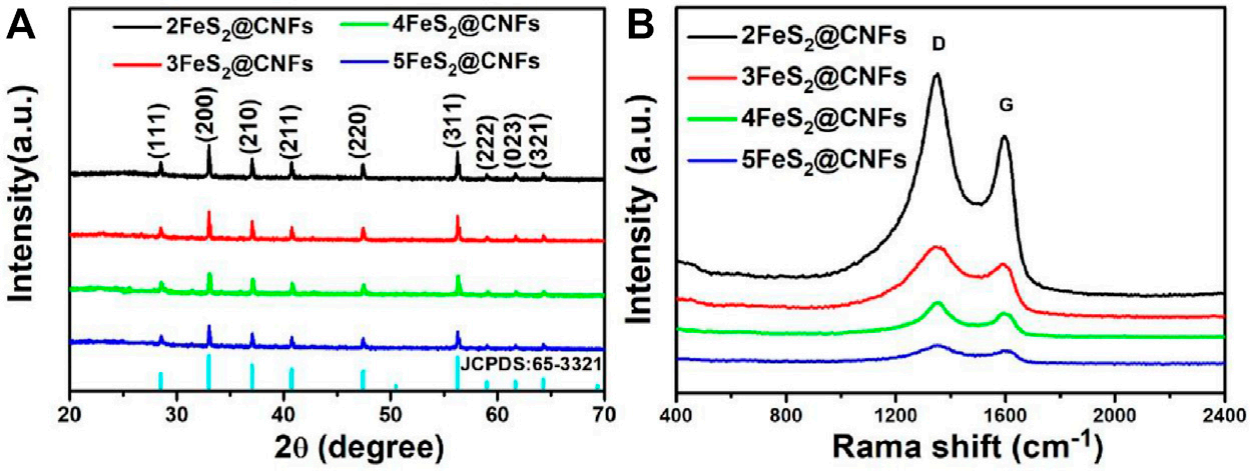
**(A)** XRD patterns and **(B)** Raman spectra.

**FIGURE 3:**
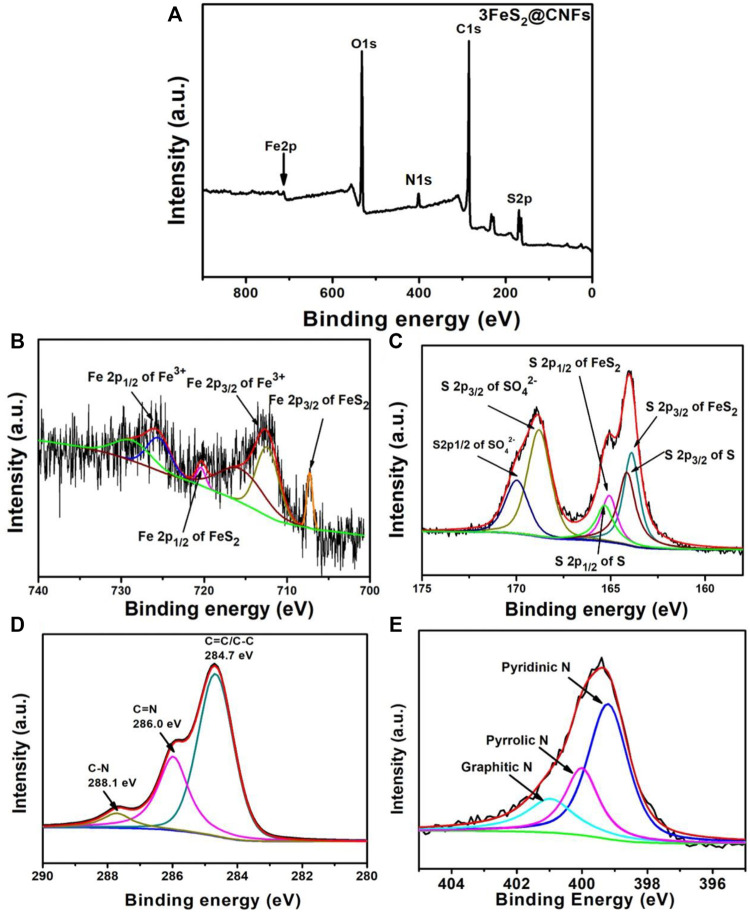
XPS of 3FeS2@CNFs: **(A)** survey spectra, **(B)** Fe 2p, **(C)** S 2p, **(D)** C1s, and **(E)** N1s.

The structures and phase purities of FeS_2_@CNFs were characterized by XRD patterns, as presented in Figure 2A. The diffraction peaks of FeS_2_@CNFs were consistent with the pure phase of pyrite FeS_2_ (JCPDS Card No. 65-3321). No diffraction peaks of the marcasite FeS_2_ and other impurities were observed. There is no diffraction peak of CNFs, indicating the formation of disordered layered graphite structures during the carbonization of PAN. This structure is composed of tiny crystals of layered graphite. The chemical composition of different FeS_2_@CNFs nanocomposites was determined using the Raman spectrum (Figure 2B). Two notable peaks at 1,352 cm^−1^ and 1,594 cm^−1^ in each spectrum match well with the D band and G band, respectively (Lu et al., 2020). The D band illustrates the defects of the carbon atom lattice, and the G band indicates the first-order scattered E_2g_ vibration mode (Xu X. et al., 2018). The ratio (I_D_/I_G_) is higher suggesting that there are more defects on the surface of CNFs (Huang et al., 2018). The values of I_D_/I_G_ for 2FeS_2_@CNFs, 3FeS_2_@CNFs, 4FeS_2_@CNFs, and 5FeS_2_@CNFs were calculated to be 1.36, 1.35, 1.33, and 1.27, respectively. As the content of FeS_2_ increases, the value of I_D_/I_G_ gradually decreases, indicating that the FeS_2_@CNFs composites transform from a disordered structure to an ordered structure. The chemical composition of 3FeS_2_@CNFs was analyzed using the XPS spectrum (Figure 3). The survey spectrum (Figure 3A) displays four typical peaks of Fe2p, O1s, C1s, and S2p, respectively. The high-resolution spectrum of Fe2p is demonstrated in Figure 3B, the two feature peaks at 707.2 and 720.3 eV belong to Fe2p_3/2_ and Fe2p_1/2_ of pyrite FeS_2_, while the two peaks at 712.3 and 725.4 eV belong to slight Fe^3+^-S or Fe^3+^-O on the surface of FeS_2_@CNFs (Chen et al., 2019). The XPS spectra of S displayed in Figure 3C are fit into six peaks. The peaks at 163.8 and 165.1 eV match well with the S2p_3/2_ and S2p_1/2_ of FeS_2_, the binding energy at 164.1 and 165.3 eV are assigned to S2p_3/2_ and S2p_1/2_ of S^2-^, and the higher binding energy at 168.7 and 169.9 eV match well with S2p_3/2_ and S2p_1/2_ of SO_4_^2-^ (Zhao et al., 2017; Lin et al., 2019). In the high-resolution spectrum of C 1s (Figure 3D), C-N, C=C/C-C, and C = N peaks are displayed (Ma et al., 2018). The production of C=N and C-N bonds is due to the addition of PAN in the electrospinning process (Huang et al., 2020). The S/N co-doped FeS_2_@CNFs can provide abundant active sites for redox reactions, improving the electronic conductivity of FeS_2_@CNFs (Lu et al., 2018).

**FIGURE 4:**
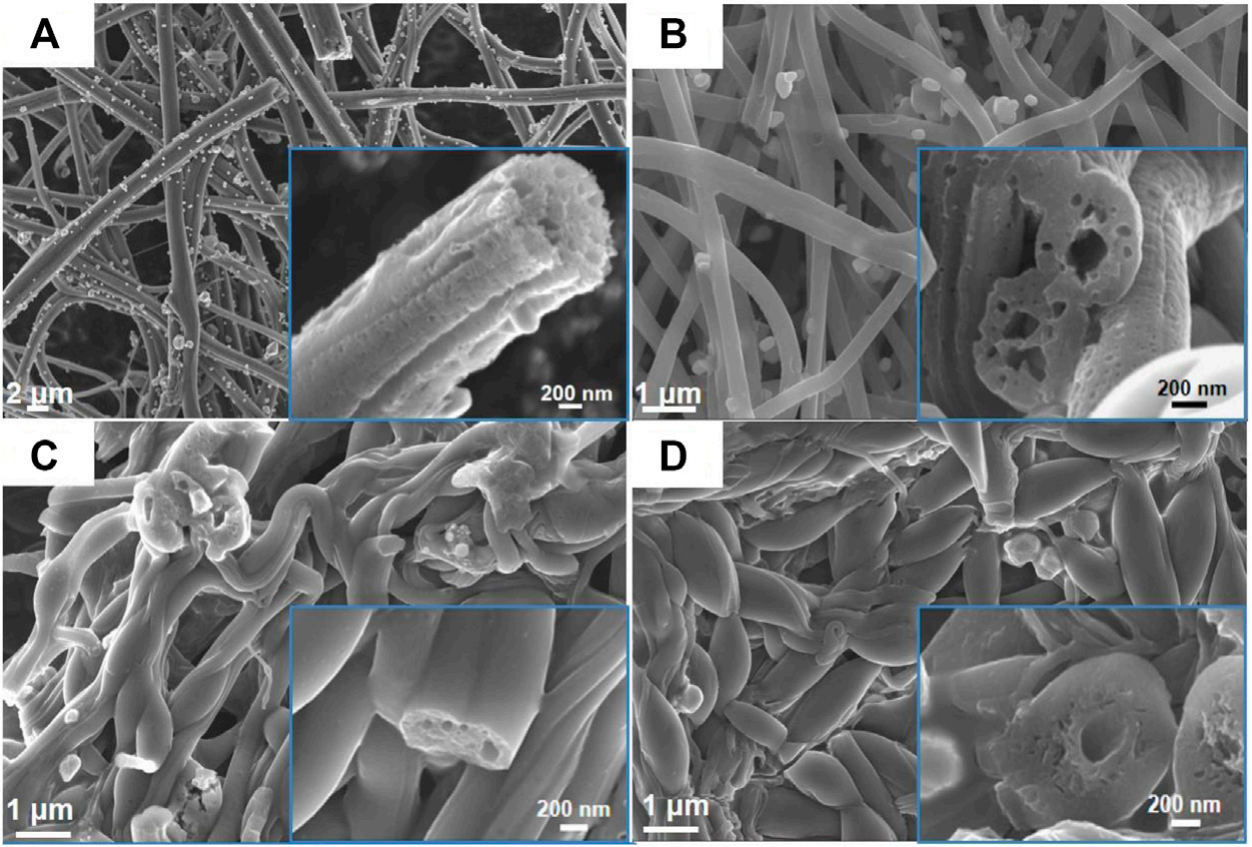
FESEM images of **(A)** 2FeS2@CNFs, **(B)** 3FeS2@CNFs, **(C)** 4FeS2@CNFs, and **(D)** 5FeS2@CNFs (The figure is a high-magnification image).

**FIGURE 5:**
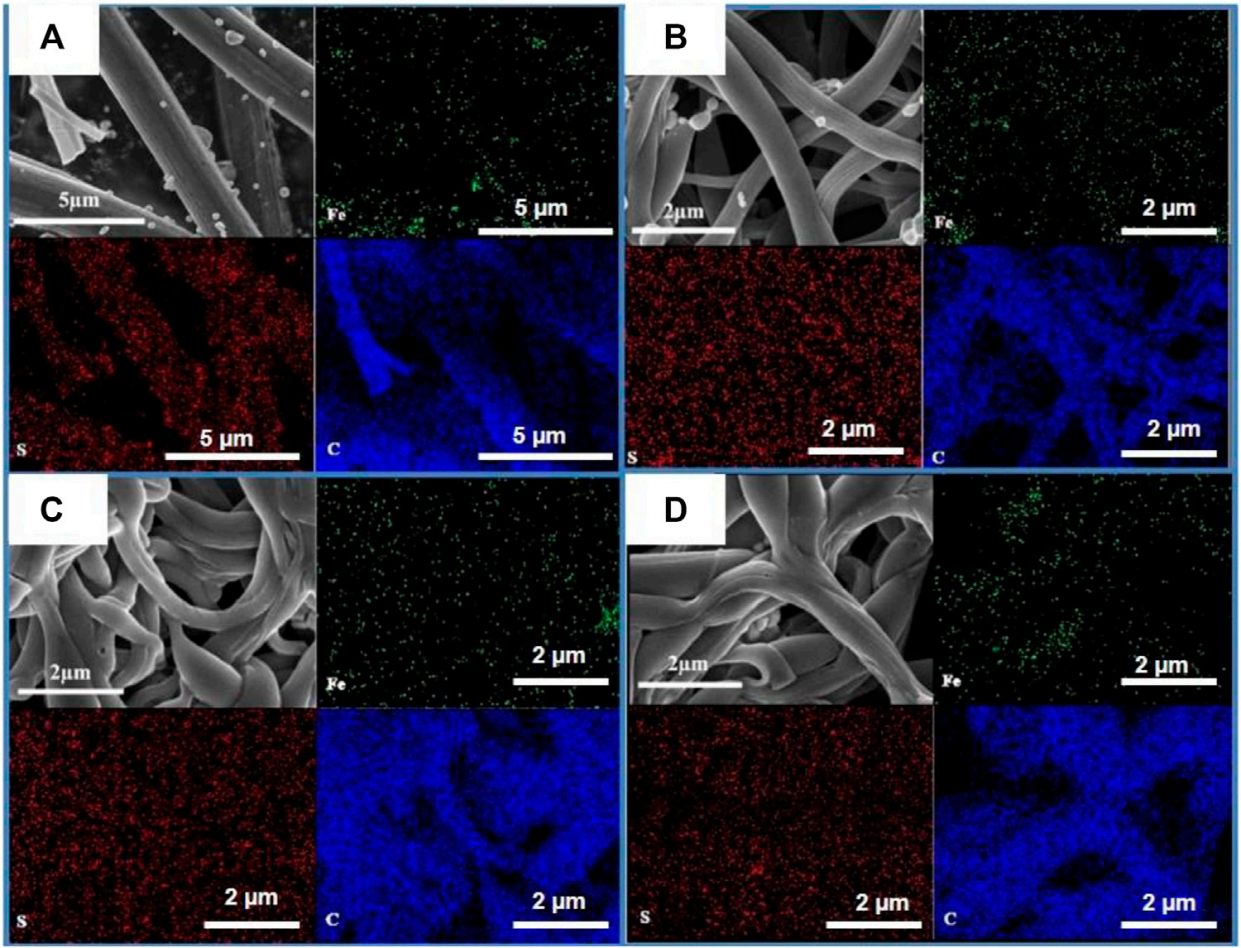
Elemental mapping and distribution of **(A)** 2FeS2@CNFs, **(B)** 3FeS2@CNFs, **(C)** 4FeS2@CNFs, and **(D)** 5FeS2@CNFs.

**FIGURE 6:**
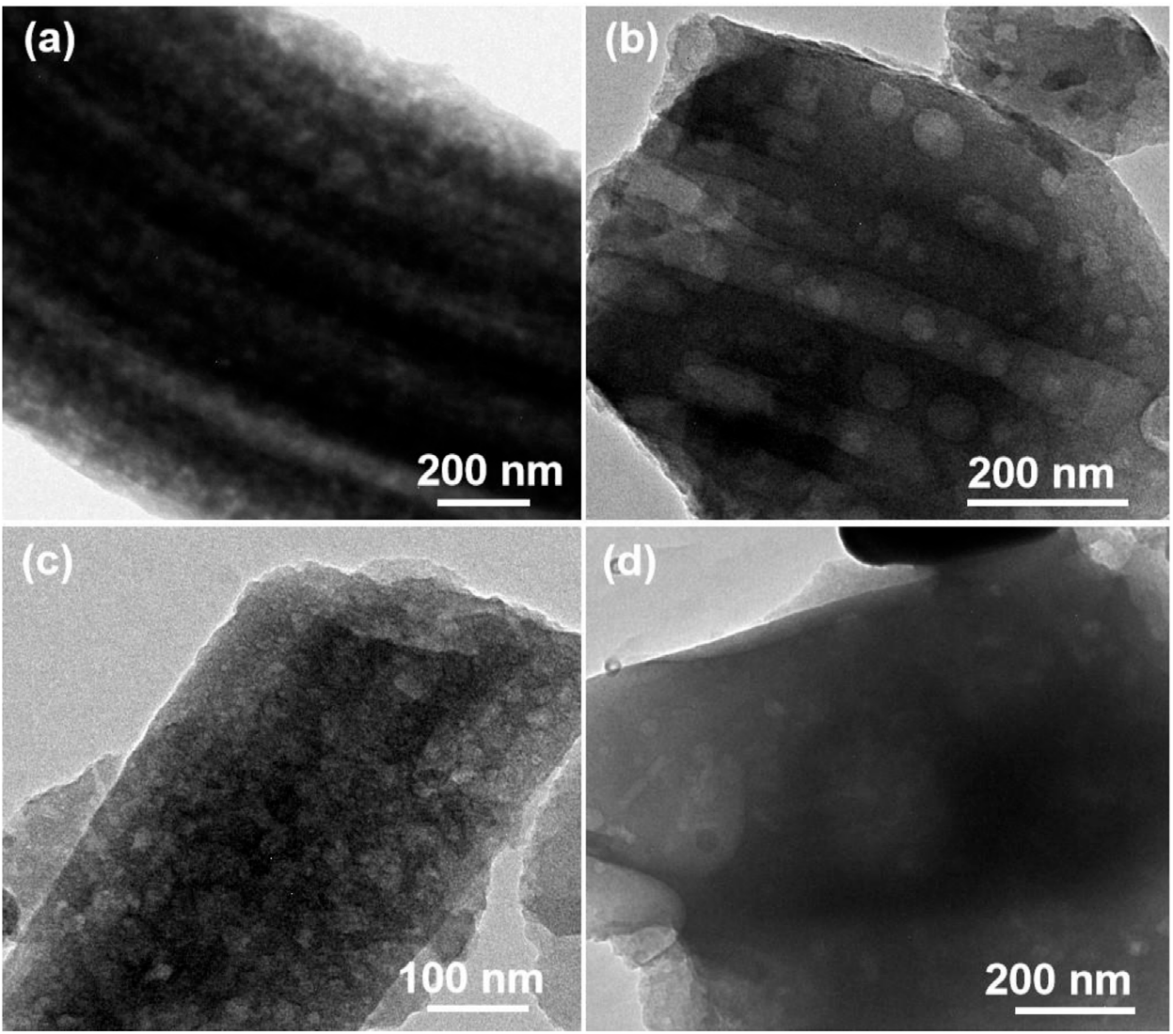
TEM images of **(A)** 2FeS2@CNFs, **(B)** 3FeS2@CNFs, **(C)** 4FeS2@CNFs, and **(D)** 5FeS2@CNFs.

The morphology characterizations of different FeS_2_@CNFs were carried out by SEM and TEM. Figure 4A displays the SEM image of 2FeS_2_@CNFs. There are many pore channels in the CNFs (the inset of Figure 4A) and Figure 5A. As the content of FeS_2_ increases to 30%, there are many pore channels with different diameters inside the nanofibers parallel to the radial direction of the nanofibers. Meanwhile, many holes appear on the surface of the CNFs, as demonstrated in Figure 4B and Figure 5B. This structure can reduce diffusion resistance and facilitate the diffusion of Li^+^ ions. At the same time, FeS_2_ nanoparticles can be firmly loaded on the inner wall of the CNFs to prevent the structure from collapsing caused by volume expansion during cycling. This multi-channel structure can also effectively prevent the dissolution of intermediate products generated during electrochemical reactions (Li et al., 2015). In the SEM and TEM images of 4FeS_2_@CNFs (Figure 4C and Figure 5C), it can be observed that the shape of CNFs becomes irregular and the phenomenon of bending and entanglement bonding appears. Furthermore, the pores inside the nanofibers are also significantly reduced. When the FeS_2_ content is 50%, the shape of CNFs is more irregular and the agglomeration phenomenon is more serious. There are no obvious pores inside the CNFs (Figures 4D, 5D). In summary, as the proportion of FeS_2_ increases, the structure of CNFs changes. This phenomenon can be attributed to the growth and aggregation of FeS_2_ particles during the reaction of iron and sulfur to form FeS_2_, occupying the space of the pores in the nanofibers. EDS measurements of the samples were investigated, as shown in Figure 6. EDS mappings present that Fe, S, C, and N are evenly distributed on their inherent positions. The Fe element originates from the addition of iron acetate during the process of experiment. The C and N elements come from PAN. The presence of S element is due to the addition of sulfur powder.

**FIGURE 7:**
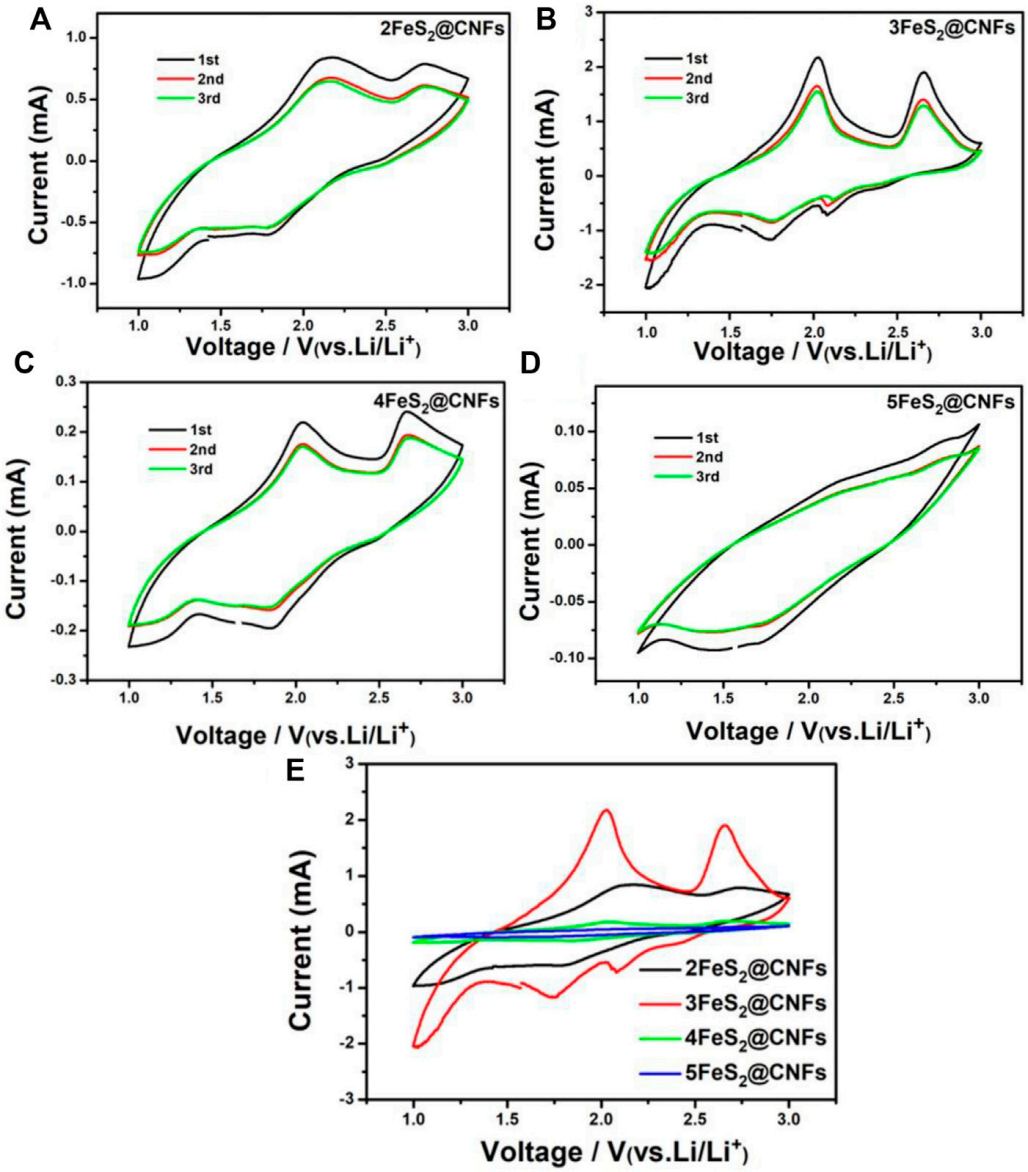
CV curves of **(A)** 2FeS2@CNFs, **(B)** 3FeS2@CNFs, **(C)** 4FeS2@CNFs, and **(D)** 5FeS2@CNFs in the initial 3 cycles at 0.5 mV s^−1^; **(E)** CV curves of the first cycle for different electrodes at 0.5 mV s^−1^.

CV is an important method to study the lithium storage behavior of FeS_2_@CNF cathodes. As shown in Figures 7A–D, CV tests were carried out for different FeS_2_@CNF cathodes at 0.5 mV s^−1^ within 1–3 V. The CV curves of 2FeS_2_@CNFs show two oxidation peaks at 2.0 and 2.6 V, and two reduction peaks at 2.1 and 1.8 V. There are two oxidation peaks at 2.1 and 2.7 V and a reduction peak at 1.8 V in the CV curves of 3FeS_2_@CNFs and 4FeS_2_@CNFs. However, no notable redox peaks can be observed in the CV curves of 5FeS_2_@CNFs. Figure 7E shows the CV curves of the first cycle for different FeS_2_@CNFs cathodes. Taking the CV curve of the 3FeS_2_@CNFs cathode as an example, the electrochemical is analyzed. The reduction peak at about 1.8 V can correspond to the below formula:

$$\mathrm{Fe}S_{2}\mathrm{+ 2L}i^{+}\mathrm{+ 2}e^{-}\to Li_{2}\mathrm{Fe}S_{2}$$ (1)

$$Li_{2}\mathrm{Fe}S_{2}\mathrm{+ 2L}i^{+}\mathrm{+ 2}e^{-}\to \mathrm{2L}i_{2}\mathrm{S + Fe}$$ (2)

The aforementioned reactions are conducted simultaneously with reaction (3). But reaction (3) can be attributed to the fact that Li^+^ ions show relatively slow diffusion in pyrite FeS_2_ at room temperature.

$$\mathrm{Fe}S_{2}+4Li^{+}\mathrm{+4}e^{+}\to \mathrm{Fe+2L}i_{2}S$$ (3)

The oxidation peak at around 2.0 V is related to the generation of Li_*2-x*_FeS_2_ according to reactions (4) and (5).

$$\mathrm{2L}i_{2}\mathrm{S + Fe}\to Li_{2}\mathrm{Fe}S_{2}\mathrm{+ 2L}i^{+}\mathrm{+ 2}e^{-}$$ (4)

$$Li_{2}\mathrm{Fe}S_{2}\to Li_{2-x}\mathrm{Fe}S_{2}+xLi^{+}+xe^{-}$$ (5)

The peak at around 2.6 V can be put down to the generation of FeS_y_ and S according to formula (6).

$$Li_{2-x}\mathrm{Fe}S_{2}\to \mathrm{Fe}S_{y}+\left(\mathrm{2-}y\right)S+\left(\mathrm{2-}x\right)Li^{+}+\left(\mathrm{2-}x\right)e^{-}$$ (6)

**FIGURE 8:**
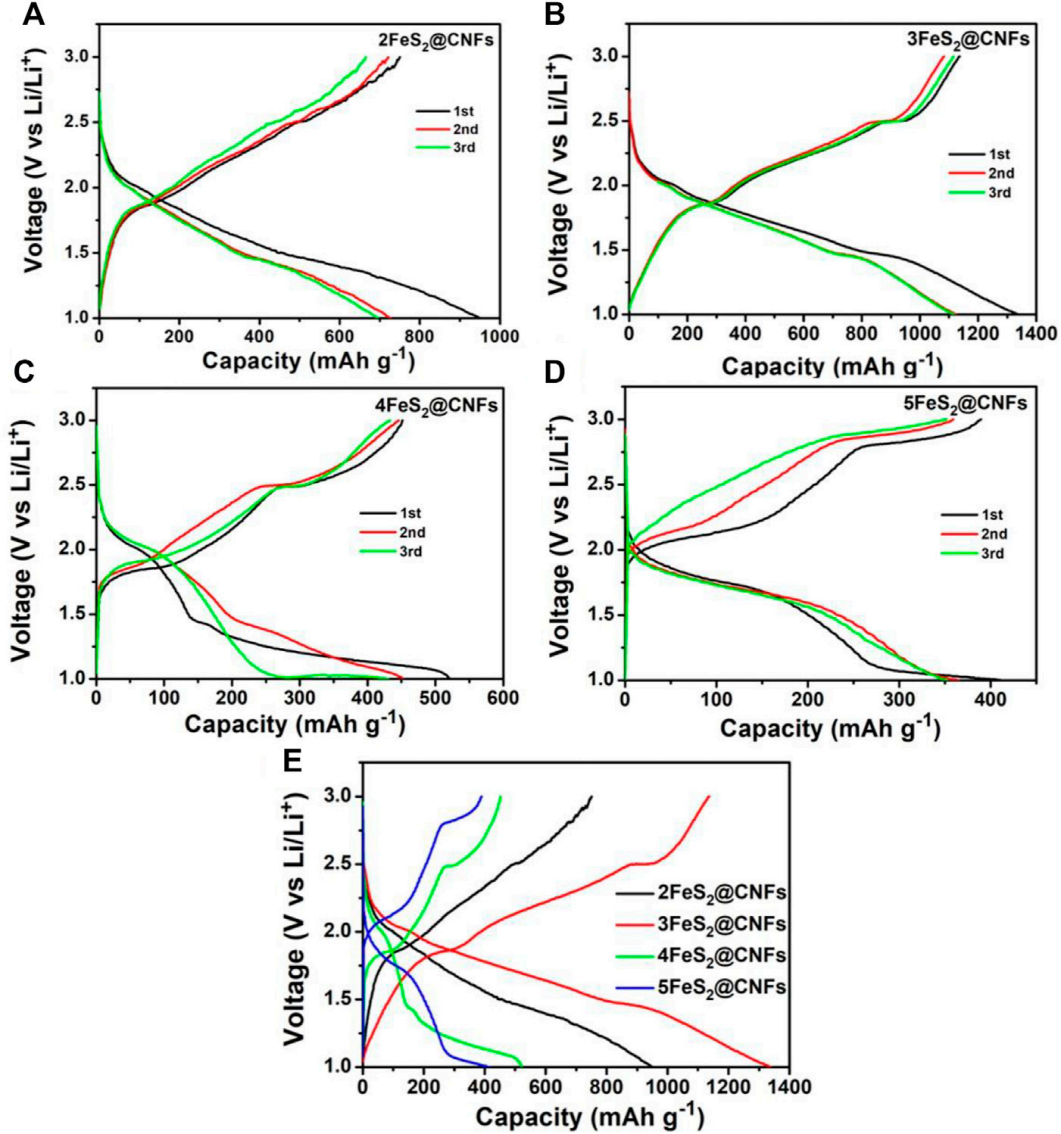
Discharge–charge profiles of **(A)** 2FeS2@CNFs, **(B)** 3FeS2@CNFs, **(C)** 4FeS2@CNFs, and **(D)** 5FeS2@CNFs 20 mA g^−1^ between 1 and 3 V; **(E)** the initial discharge-charge profiles of different electrodes at 20 mA g^−1^.

Figures 8A–D are the GCD curves of the first three circles for different FeS_2_@CNFs cathodes at 20 mA g^−1^. It can be observed that the charge and discharge platforms of each cathode are matched well with the CV curves. Figure 8E shows the GCD profiles of the initial cycle for different cathodes at 0.02 A g^−1^. The initial discharge capacity of 2FeS_2_@CNFs, 3FeS_2_@CNFs, 4FeS_2_@CNFs, and 5FeS_2_@CNFs is 905.8, 1,336.7, 520.3, and 400.9 mAh g^−1^, respectively. It is obvious that 3FeS_2_@CNFs composites show a relatively high specific capacity. This is mainly because CNFs can not only improve the conductivity of the electrodes, but its internal pores can also facilitate the reversible embed/de-embed of Li^+^ ions. In addition, FeS_2_ nanoparticles can be uniformly distributed in the pores, increasing the contact area between the FeS_2_ and Li^+^ ions, and effectively prevent the dissolution of polysulfides generated during the discharge process (Li et al., 2020). 2FeS_2_@CNFs also have many pores, but the content of FeS_2_ is relatively low, so the specific capacity is less than that of 3FeS_2_@CNFs. As the content of FeS_2_ increases, the resistance of 4FeS_2_@CNFs and 5FeS_2_@CNFs increases, so their specific capacitances decrease.

**FIGURE 9:**
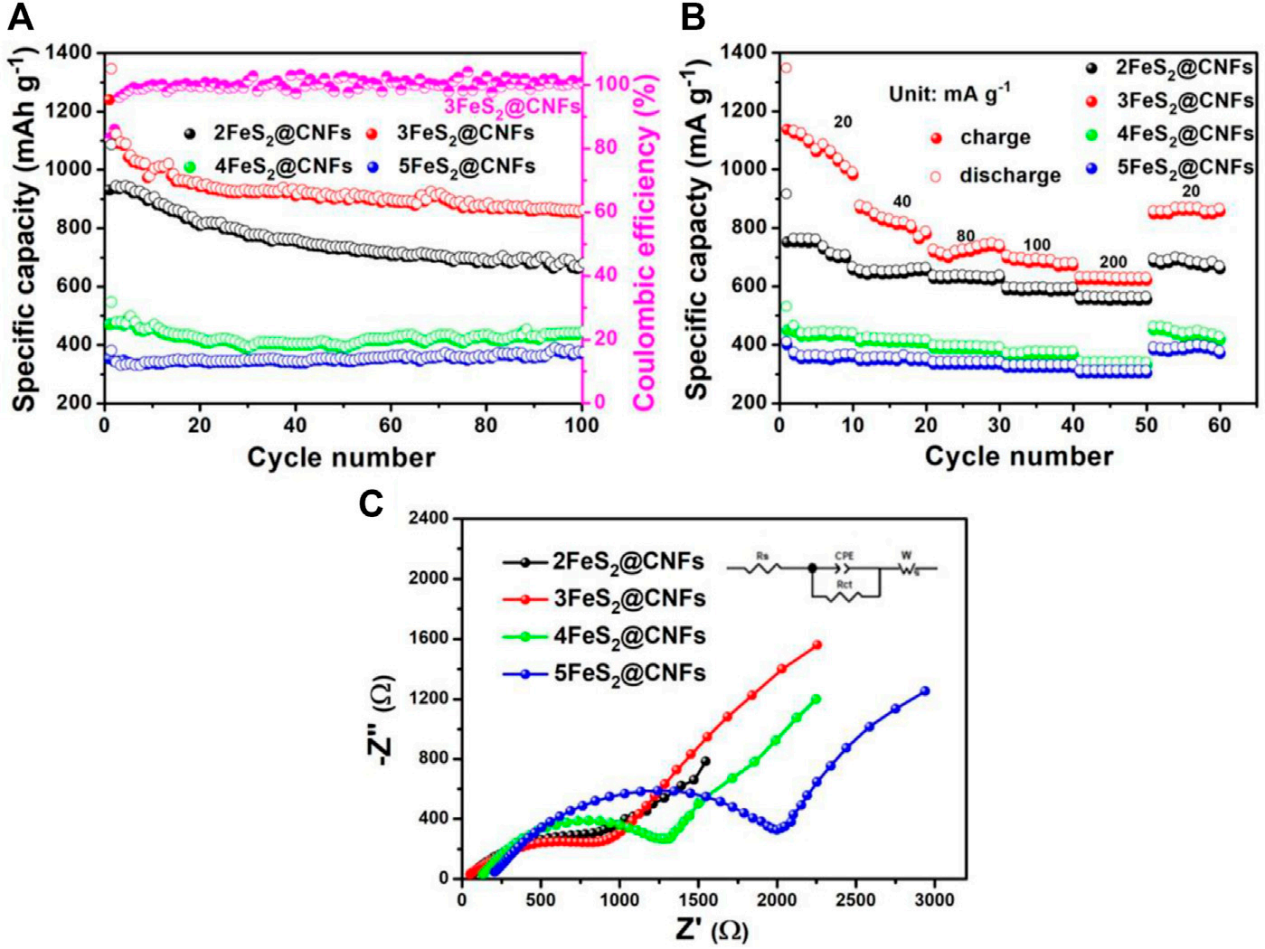
**(A)** Cycling performance at 20 mA g^−1^, **(B)** rate performance, and **(C)** EIS plots of the different electrodes.

The cyclic performance of the samples was also determined, as presented in Figure 9A. The specific capacity of 2FeS_2_@CNFs, 3FeS_2_@CNFs, 4FeS_2_@CNFs, and 5FeS_2_@CNFs is 674.6, 856.5, 440, and 370 mAh g^−1^ at 20 mA g^−1^ after 100 cycles. The specific capacities of 2FeS_2_@CNFs and 3FeS_2_@CNFs decay during the cycling, which can be attributed to the dissolution of polysulfides during the electrochemical reaction and leading to the loss of active materials. As the proportion of FeS_2_ increases, the FeS_2_ in the pores of carbon fibers can build up and agglomerate. Therefore, the space of the pores becomes less and less, resulting in a reduction in the contact area between Li^+^ ions and active materials so that the phenomena of 4FeS_2_@CNFs and 5FeS_2_@CNFs are not obvious. Figure 9B shows the rate performance curves of different samples at 20, 40, 80, 100, and 200 mA g^−1^, respectively. It is evident that the 3FeS_2_@CNFs cathode exhibits the highest rate performance at various current densities among the four cathodes. The excellent electrochemical performances of the 3FeS_2_@CNF cathode can be attributed to the multi-channel structure of CNFs, which can supply abundant paths for ion and charge transfers. The EIS of 2FeS_2_@CNFs, 3FeS_2_@CNFs, 4FeS_2_@CNFs, and 5FeS_2_@CNFs was confirmed, as displayed in Figure 9C. The values of the equivalent series resistance and the charge transfer resistance for 3FeS_2_@CNFs are the smallest. The results indicate that the ratio of FeS_2_ and CNFs is appropriate, which allows the cathode materials possess more three-dimensional hollow channels. Therefore, numerous paths are provided to promote the transport of Li^+^ ions and electrons, improving the electroconductivity of the cathodes.

## 4 Conclusion

In summary, the novel FeS_2_@CNFs nanocomposites with the multi-channel structure are successfully prepared by the electrospinning method. The 3FeS_2_@CNFs cathode exhibits an admirable capacity of 856.5 mAh g^−1^ at 20 mA g^−1^ after 100 cycles. The excellent electrochemical properties can be attributed to the right ratio of FeS_2_ and carbon nanofibers that can produce lots of hollow channels. The three-dimensional interlinked multi-channel carbon nanofibers can facilitate the diffusion of Li^+^ ions and electrons, improving the electroconductivity of cathodes. Meanwhile, the FeS_2_ nanoparticles are distributed on the inner wall of the carbon nanofibers, improving the phenomenon of the volume expansion for FeS_2_ and preventing the dissolution of polysulfides during the cycling process. In addition, S/N co-doped FeS_2_@CNFs can supply abundant active sites for electrochemical reactions, providing enough space for Li^+^ ion storage. Thus, the as-prepared 3FeS_2_@CNFs are a splendid cathode material for lithium-ion batteries, and it can be one of the promising candidates for next-generation secondary batteries.

## Data Availability

The original contributions presented in the study are included in the article/Supplementary Material; further inquiries can be directed to the corresponding author.
